# Identification of low oxygen-tolerating bacteria in prostate secretions of cancer patients and discussion of possible aetiological significance

**DOI:** 10.1038/s41598-017-13782-6

**Published:** 2017-11-09

**Authors:** Roshni Bhudia, Amar Ahmad, Onyinye Akpenyi, Angela Whiley, Mark Wilks, Tim Oliver

**Affiliations:** 1 Barts and The London School of Medicine and Dentistry, Queen Mary University of London, E1 2AD, London, UK; 2 Wolfson Institute for Preventive Medicine, Barts and The London School of Medicine and Dentistry, Queen Mary University of London, EC1M 6BQ, London, UK; 3 Microbiology, Immunobiology, Blizard Institute, Barts and The London School of Medicine and Dentistry, Queen Mary University of London, E1 2AD, London, UK; 4 Oncology, Barts Cancer Institute, Queen Mary University of London, EC1M 6BQ, London, UK

## Abstract

The microaerophylic organism *Propionibacterium acnes* has shown consistent association with prostate cancer (PC). Studies linking circumcision with reduced PC further support anaerobes involvement as circumcision reduces anaerobe colonisation on the glans penis. A 1988 study linked anaerobes with PC but considered them as opportunists in necrotic tumour. A hypothesis that a “*Helicobacter*-like” process causes PC justified this pilot study. Active surveillance patients were enrolled. Post-prostate massage urine samples were screened using the Matrix-Assisted Laser Desorption/Ionization Time-of-Flight (MALDI-TOF) technique for bacterial identification after culture in anaerobic and aerobic conditions. 8 out of 18 patients (41%) had either obligate anaerobic (n = 5) or microaerophilic (n = 4, one of whom also had anaerobes) organisms identified. None of 10 control samples contained obligate anaerobes. Although mean PSA was 63% higher in those with low oxygen tolerating bacteria, two high outliers resulted in this difference being non-significant. Given the substantially higher proportion of PC patients with organisms growing in a low concentration of oxygen when combined with previous studies compared to controls, the degree of significance was as high as smoking 5–9 cigarettes a day and needs further investigation. Translational research in trials combining Vitamin D and aspirin have begun as part of such investigation.

## Introduction

There is considerable amount of evidence that chronic inflammation is a concomitant of cancer development in most sites, including prostate cancer^1^. It is unclear whether it is a precursor/promotor of cancer development^2,3^, just a manifestation of a response to the disorderly development that occurs as the cancer progresses^4^ or a mixture of both as has been suggested by the hypothesis of cancer as a wound that does not heal^5,6^. Linkage between low oxygen-tolerating organisms and malignant transformation has been established with evidence for the association of the microaerophylic bacterium, *Helicobacter.pylori*, in stomach cancer^7,8^. More limited is evidence that Vitamin D deficiency is a promoting factor for this cancer type^9,10^. In prostate cancer there is no well accepted pathogen consistently associated with this malignancy nor the inflammation associated with it despite multiple studies, many of which have provided early suggestions but failed to be confirmed on subsequent studies^11^.

However, recent studies have suggested that infection with *Propionibacterium.acnes* (PA), a known sun-sensitive^12^ microaerophylic bacterium associated with acne, has shown a more consistent association with prostate cancer^13–18^. Studies linking circumcision with reduced risk of prostate cancer^19–21^ is further supportive evidence of the role of such organisms in prostate cancer aetiology as randomised trials of circumcision have demonstrated reduced anaerobe colonisation of the glans penis after circumcision^22^. The uncircumcised glans penis has direct access to the prostate through the urethra^23^ which is relatively anoxic. Although there is one report from 1988 linking anaerobes with prostate cancer^24^ and one study demonstrating that antibiotics can clear them from the prostate^25^, most workers have until recently considered them as opportunists colonising necrotic tumour tissue rather than being considered as actual causative factors. Due to a long-held view that a “*Helicobacter*-like” process could be involved in causation of prostate cancer^26–29^, this pilot study was undertaken to screen for aerobic and anaerobic bacteria by culturing small aliquots of urine samples obtained following a “mini-prostate massage” done for PCA3 testing. The use of a commercial MALDI-TOF (Matrix-Assisted Laser Desorption/Ionization Time-of-Flight) mass spectrometry device allowed for the rapid and accurate identification of bacteria.

## Results

### Clinical demographics of participants

Table 1 lists age, clinical and Gleason Grading pathology staging and PSA levels of 18 patients selected for study while undergoing a period of surveillance without having had any surgical, radiation or drug treatment. Table 2 lists the diagnosis in the group of non-prostate cancer controls undergoing microbiological urine testing for non-malignant causes who were selected for having adequate surplus urine for anaerobic culture.

**Table 1:** Clinical characteristics of prostate cancer patients.

**Specimen Identifier**	**AGE**	**PSA**	**T stage**	**Gleason Grade**
01 ML	58	1.29	T1, N0, M0	G 3 + 3
02 PH	57	4.54	T2, N0, M0	G 3 + 3
03 PO	60	7.3	T1, N0, M0	G 3 + 3
04 HS	72	6.1	T2, N0, M0	G 3 + 3
05 FJD	63	5.08	T2, N0, M0	G 3 + 3
06 PK	74	10.7	T3a, N0, M0	G 3 + 4
07 PW	69	3.22	T1, N0, M0	G 3 + 3
08 JL	69	12	T2, N0, M0	G 3 + 4
09 CO	55	6.7	T2, N0, M0	G 3 + 4
10 SP	66	12	T1, N0, M0	G 3 + 3
11 AH	69	11	T1, N0, M0	G 3 + 3
12 ST	62	26	T2, N0, M0	G 4 + 5
13 PL	61	7.4	T3a, N0, M0	G 4 + 3
14 AB	75	9.52	T1, N0, M0	G 3 + 4
15 AO	52	4.84	T1, N0, M0	G 3 + 3
16 WP	85	26	T2, N0, M0	G 3 + 4
17 SR	65	5.36	T1, N0, M0	G 3 + 3
18 KP	69	7.5	T1, N0, M0	G 3 + 3

**Table 2:** Control “normal” males.

**Specimen Identifier**	**Age**	**Diagnosis**
01 MA	85	Under investigation
02 OSt-L	77	Pre-op
03 EC	81	Routine blood
04 MC	80	Pre-op
05 RP	77	Respiratory arrest
06 MLu	70	Post op
07 BM	87	Under investigation
08 RS	76	A&E attendance
09 GA	65	ITU
10 KD	72	Cardiac arrest

### Oxygen dependence of organisms detected after prostate massage in prostate cancer patients

Table 3 summarises microbiology findings and shows that 5 of 18 (27.8%) had obligate anaerobe (4/5 having *Peptoniphilus harei*) and 4 (1 of who also had pure anaerobe) of 18 (22%) had microaerophylic bacteria, *i.e*., 8/18 (44%) had one or other type of low oxygen-tolerating organisms in post-prostate massage urine. This compares to 0 of 10 “normals” having obligate anaerobes.

**Table 3:** Oxygen dependence of organisms detected after prostate massage in study patients.

**Patient code**	**DATE**	**Aerobic and facultatively anaerobic Organisms isolated**	**Obligate anaerobic Organisms isolated**	**Microaerophylic Organisms isolated**
01 ML	6/8/15	Corynebacterium amycolatum Staph haemolyticus	Nil	Nil
02 PH	6/8/15	Strep sp, Staph haemolyticus & C. glucuronydictum	Peptoniphilus harei Veillonella montpenellerensis	Nil
03 PO	10/8/15	Strep sp, Staph hominis & Dermabacter hominis	Nil	Actinomyces neuii
04 HS	11/8/15	Aerococcus urinae, staph epidermidis, Staph simulans Corynebacterium tuberculostearicum	Fusobacterium nucleatum Fusobacterium gondiaformans Peptoniphilus harei Actinobaculum schaali?i	Nil
05 FJD	27/8/15	Nil	Nil	Nil
06 PK	27/8/15	Enterococcus faecalis	Nil	Nil
07 PW	3/9/15	Staph epidermidis & Staph haemolyticus	Nil	Nil
08 JL	27/8/15	Enterococcus faecalis, Staph epiderm & Micrococcus luteus	Nil	Brevibacterium casei
09 CO	10/9/15	Nil	Nil	Nil
10 SP	10/9/15	Strep agalactiae, Gardnerella sp Strep pneumoniae, Strep anginosus	Nil	Nil
11 AH	10/9/15	Staph epiderm, Staph hominis Dermabacter hominis C. tuberculostearicum	Nil	Nil
12 ST	10/9/15	Staph capitis, Staph hominis Strep anginosus Acinetobacter radioresistens	Peptoniphilus harei	Nil
13 PL	10/9/15	C. tuberculostearicum Strep agalactiae, Strep angino Dermobacter hominis	Veillonella parvula Actinobaculum schaalii	Actinomyces turicencis Brevibacterium paucvorans
14 AB	17/9/15	Staph epiderm, *Citrobacter koseri* Strep pneumoniae	Nil	Nil
15 AO	17/9/15	Staph hominis Staph haemolyticus Strep anginosus	Nil	Nil
16 WP	17/9/15	C. amycolatum Strep anginosus Dermobacter hominis Staph epidermis	Peptostreptococcus anaerobius, Peptoniphilus harei Finegoldia magna	Nil
17 SR	17/9/15	Aerococcus urinae, E. faecalis Staph haem, Staph hominis, M luteus, Strep pneuminiae Kocuria rhizophilia	Nil	Actinomyces neuii
18 PK	17/9/15	Staph epidermidis, Staph capitis, Staph haemolyticus E. faecalis	Nil	Nil

### PSA and type of bacterial isolate

**Figure 1:**
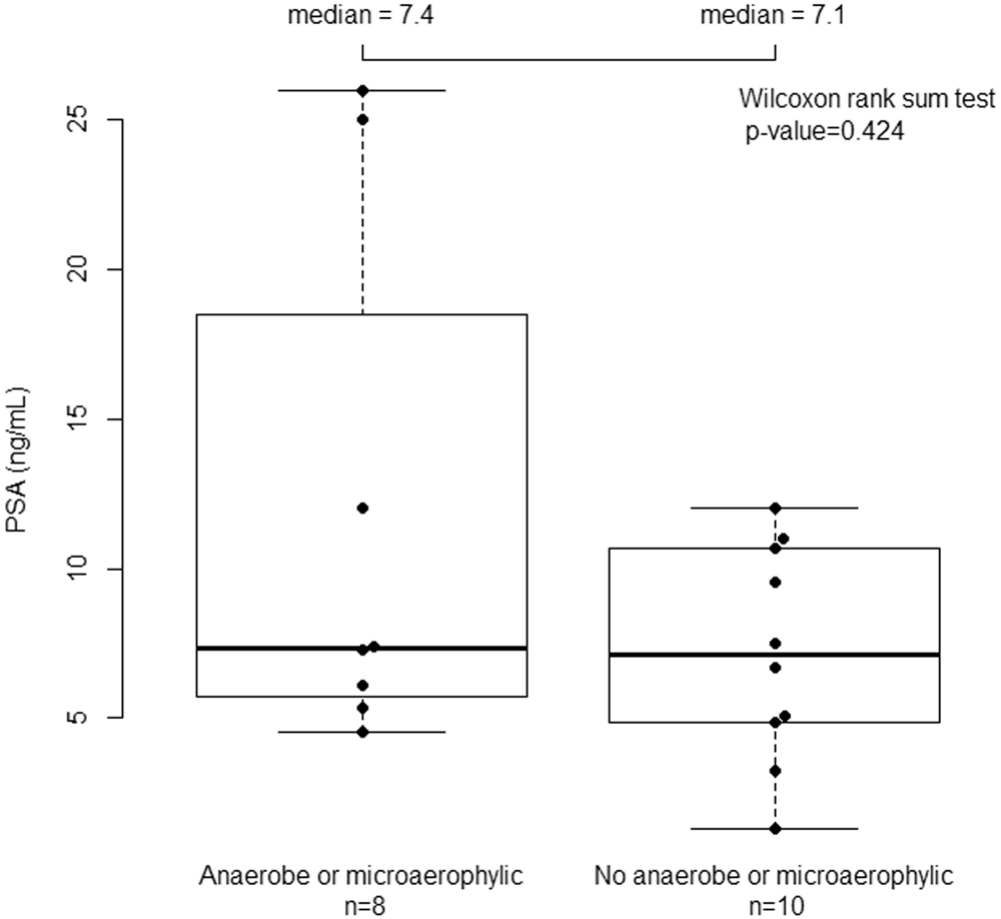
Shows a dot plot distribution of PSA in 18 Active Surveillance patients comparing combined group of obligate anaerobe & microaerophilic positive patients and those without such bacteria.

Figure 1 shows results of dot plot distribution comparing the mean PSA of those urines containing obligate anaerobe or microaerophylic bacteria (mean PSA 11.7 range 4.5–26) and those with no such organism (mean PSA 7.2 range 1.29–12). Although the mean PSA level was 63% higher in the patients with low oxygen-tolerating bacteria, the small numbers and two high outliers of 26 ng/m (both positive for Peptoniphilus harei) result in the difference in the medians only being 7.4 vs 7.1 and the distribution being non-significant in Wilcoxon rank sum test (p value = 0.423).

## Discussion

It has been known since the study of Cooper *et al*. 1988^24^ that anaerobes are present more frequently in the tissue biopsies of malignant than benign prostates and that this frequency was higher than contemporary reports of urine cultures of normal subjects^30^. Recently there has been a cluster of reports showing a higher frequency of the microaerophylic organism *P. acnes* in malignant compared to benign prostates^13–18^ and reports that circumcision both reduces anaerobe contamination of the glans^22^ and reduces the risk of prostate cancer^19–21^. As a consequence, interest in both circumcision and these classes of bacteria has increased. This is particularly so since the cohort study of prostate cancer risk and circumcision by Spence *et al*.^20^. This study involved 1590 French Canadian residents and 1618 electoral registry controls. Although it failed to show any significant protection in the majority white population (OR 0.93 95% CI 0.80–1.12) except in men circumcised after the age 36 and non-significantly in the 26% of their men circumcised at birth (OR 0.86 95% CI 0.72–1.04), there was significant reduction (OR 0.44 95% CI 0.19–0.86) in the small subgroup of black men. More significant in respect of the results of our study was the fact that although there was no association in Spence *et al*’s study with history of an STD, there was an association with a history of prostatitis. Perhaps the most important observation from this study was the observation that circumcision after the age of 36 gave protection. Clearly as an unpredicted observation it needs to be repeated but based on the observation contained in our report and the evidence on the role of phimosis in development of penile cancer^31^, the easiest way to recruit people to a study that clarified whether anaerobes do contribute to the raised PSA would be to target patients with raised PSA presenting with phimosis and measure PSA before and after surgery.

Although our study is small and lacks adequate matched contemporaneous controls, these preliminary results certainly are different from those obtained from normal urine by others using earlier techniques^30^ as well as our small series of “normal” controls, admittedly without prostate massage. However, pooling this limited literature data base of 27 prostate cancers and 551 “controls” (Table 4) produces a highly statistically significant difference (Pearson's χ² = 70.304 (df = 1, p < 2.2e-16) with Yates' continuity correction), providing justification for a prospective study including age- and sex-matched control samples from routine health care procedures such as cardiac stents and hip replacements and patients with benign prostatic hyperplasia (BPH).

**Table 4:** Summary reports of anaerobes in normal urines & tissue from patients with BPH vs with prostate cancer.

	**No of cases studied**	**Positive anaerobes**
Normal Urine^30^	517	5.9%
BPH tissue #^24^	24	0%
Control “normal” urines (Bhudia *et al*. this series)	10	0%
Prostate Cancer tissue ##^24^	9	67% # vs ## P = 0.001 Fishers exact test
Prostate Cancer urines (Bhudia *et al*. this series)	18	44%
TOTAL “CONTROLS”	551	5.5%
TOTAL PROSTATE CANCER	27	52% Pearson's χ² = 70.3 (df = 1, p < 2.2e-16) with Yates' continuity correction

In this small number of cases studied, no case was found with either *P. acnes* or *H. pylori* (the microaerophylic organism proven to have malignant association with stomach cancer^2^) nor the facultative anaerobe, *Streptoccocus gallolyticus*, (associated with colon cancer^32,33^) although there was one case (Tables 3, 4) associated with one of the *Fusobacterium species*, an anaerobe recently reported to be associated with pancreatic cancer^34^. Given the increasing numbers of cancers reported to have an association with anaerobes, they do give more support for the concept that, given the known hypoxic state of cancers, such organisms could indeed play a role either accelerating progression^35^ or in addition playing a role in the induction as proven in stomach cancer. In this respect prostate cancer is possibly a particularly informative cancer to begin study of the mechanism involved given the data demonstrating reduced long-term sun exposure having a more clear-cut association (Table 5) with increased risk of cancer^28,36,37^, than spot Vitamin D levels^38^. As puberty is a time when males prove particularly sensitive to *P. acnes* and as with breast and cervix cancer there is some evidence linking early puberty with prostate cancer risk^27,39^, it could be that population-based studies of PSA levels in pubertal males^29^ might give further evidence that could go some way to support the hypothetical interpretation of these data. This is that a 40–60 year life time of urban living with sub-clinical Vitamin D-induced, macrophage-mediated, non-antigen-specific immune deficiency^40^ is as oncogenic in terms of the non-smoking related cancers as smoking between five to nine cigarettes a day over 40–60 years is a cause of lung cancer^41^ (Table 5). An even more fruitful area for research is suggested by the observation of Head *et al* (Table 5) who demonstrated a 2.5 odds ratio for increased death from cancer at 10–15 years of follow up of civil servants taking sick leave for a psychiatric cause^42,43^. Many such people would be likely to rise with the sun, return home at sunset, take lunch at their desks and suffer from “non-seasonal-SAD” (seasonal affective disorder) because, whatever sun they were exposed to, did not follow the normal seasonal variation. Further population-based studies of “CBT-light” in such patients^44^ could be very informative with serial monitoring of mood and anonymised screening of the bloods of recruited patients for PSA. Clearly such a study (limited to adults over the age of 40 given data that such individual with PSA greater than the median level of age corrected PSA had a 3.75 HR of developing prostate cancer^45^) would only be justified if a non-surgical approach had been shown to normalise PSA long-term.

**Table 5:** Comparison of anaerobes and prostate/cervix ca risk and imprecise measures of sun exposure vs more precise lung ca risk of low dose tobacco.

	**No of studies**	**Actual or Median level of risk**
Geographic study of PC risk low vs high sun exposure^55^	8	1.36 (range 1.01–1.73)
Clinical Questionnaire PC risk low vs high sun exposure^55^	8	1.13 (range 1.0–1.41)
Bacterial vaginosis & Ca Cervix^56^	19	1.51 (1.24–1.83)
HPV, BV and abnormal cytology vs BV alone vs BV absent^57^	1	3.82 vs 2.91 vs 1.00
10 yr cancer mortality post a psychiatric sick note vs no note in civil service^58^	1	2.49 (1.33 to 4.68)
Male death Lung cancer from smoking 1–4 cigarettes a day^59^	1	2.79 ((0.94 to 8.28)
Presence of anaerobes in prostate & PC risk see Table 4 for actual data	1	Odds ratio* (95% C.I.) = 17.8 (7. 6, 42.0); p-value= 3.88e-10 * Odds ratio was computed by median-unbiased estimation (exact CI).
Male death Lung cancer from smoking 5–9 cigarettes a day^59^	1	11.1 (6.94–20.44)

Given laboratory studies in mice that demonstrated that this class of bacteria can be controlled therapeutically by treatment with Vitamin D^46^ and the continuing amounts of data supporting the link between Vitamin D or sun exposure deficiency with increased risk of human cancer, trials of Vitamin D combined with anti-inflammatory agents such as aspirin aiming to reduce tumour progression (see http://www.isrctn.com/ISRCTN91422391 and https://www.provent.org.uk) could provide evidence to support the above interpretation. The increasing recognition of the role of Vitamin D in enhancing non-antigen-specific macrophage anti-pathogen responses^40,47^ provides an understandable mechanism. However, given the similarities in bacterial flora between what we have found and those in bacterial vaginosis, recently increasingly accepted as promoting HPV-induced cervix cancer (Table 5)^48,49^, it is likely for long-term control that treatment of both the patient and his partner may be necessary with selective use of appropriate anti-anaerobe antibiotic if response does not occur to Vitamin D alone. However, given the increasing problems with widespread antibiotic resistance and recent reports of successful use of *E. coli* vaccines in women with recurrent urinary tract infection^50^, it is likely that such a non-antibiotic approach may be preferable^51^.

To conclude, MALDI-TOF enabled rapid and accurate identification of bacteria present in the post-prostate massage urine. A higher proportion of organisms growing in a low concentration of oxygen were found in prostate cancer samples than in previous normal studies and in the small number of “control” urines tested.

Given the association of cervical cancer with bacterial vaginosis and increased knowledge about how organisms such as *Helicobacter pylori* induce stomach cancer and association of *Streptoccocus gallolyticus* with colon cancer, larger scale studies in prostate cancer patients are required using techniques such as MALDI-TOF or Metagenomic sequencing^52^ to rapidly identify bacteria which have hitherto been problematic to identify.

Given the increasing number of indications that the first event in the initiation of prostate cancer may be occurring in the early years of puberty, there is a need to find ways to better assess annual sun exposure to quantify the effect of the 30–40 years of chronic sun deficiency in modern urban environments.

## Methods

### Patient selection

The patients selected attended the prostate cancer clinic at Barts Hospital (Barts Health NHS Trust) and were patients on follow up or planned for active surveillance. Two medical students as part of an approved audit (ref no. 6227) identified and collected clinical and biochemical information of suitable patients. The consultant in charge took informed consent for donation of post-prostate examination urine collection under City & London East ethics approval (ref no. 09/H0704/4+5 date 16/07/2014). The students then undertook delivery to the laboratory and observed the screening procedure which was undertaken in microbiology in accordance with the approved protocol. In addition, a selection of surplus urines from routine urine requests from age-matched men, were screened for obligate anaerobes only as well, although they had not had prostate massage.

### Microbiology

1.5 ml of urine was centrifuged at 16,000 g for 2 minutes. The supernatant was then discarded and 10 ul of the deposit plated on Columbia Horse Blood agar (Thermofisher, Basingstoke, UK) and Fastidious anaerobic agar (Thermofisher, Basingstoke, UK). Plates were incubated for 72h at 35˚C in air + 5% CO_2_ or anaerobically in an atmosphere of 90% N2, 5% H2 and 5% CO2. Resultant colonies were identified by MALDI-TOF using a Bruker Maldi Biotyper mass spectrometer^53,54^. All data generated and analysed during this study are included in this published article.
